# Growth Pattern Responses to Photoperiod across Latitudes in a Northern Damselfly

**DOI:** 10.1371/journal.pone.0046024

**Published:** 2012-09-21

**Authors:** Szymon Śniegula, Viktor Nilsson-Örtman, Frank Johansson

**Affiliations:** 1 Department of Ecosystem Conservation, Institute of Nature Conservation, Polish Academy of Sciences, Kraków, Poland; 2 Department of Ecology and Environmental Science, Umeå University, Umeå, Sweden; 3 Department of Ecology and Genetics, Uppsala University, Sweden; California State University Fullerton, United States of America

## Abstract

**Background:**

Latitudinal clines in temperature and seasonality impose strong seasonal constraints on ectotherms. Studies of population differentiation in phenotypic plasticity of life history traits along latitudinal gradients are important for understanding how organisms have adapted to seasonal environments and predict how they respond to climate changes. Such studies have been scarce for species with a northern distribution.

**Methodology/Principle Finding:**

Larvae of the northern damselfly *Coenagrion johanssoni* originating from semivoltine central, partivoltine northern, and partivoltine northernmost Swedish populations were reared in the laboratory. To investigate whether larvae use photoperiodic cues to induce compensatory growth along this latitudinal gradient, larvae were reared under two different photoperiods corresponding to a northern and southern latitude. In addition, field adult size was assessed to test the strength of possible compensatory growth mechanisms under natural conditions and hatchling size was measured to test for maternal effects. We hypothesized that populations originating from lower latitudes would be more time constrained than high-latitude populations because they have a shorter life cycle. The results showed that low-latitude populations had higher growth rates in summer/fall. In general northern photoperiods induced higher growth rates, but this plastic response to photoperiod was strongest in the southernmost populations and negligible in the northernmost population. During spring, central populations grew faster under the southern rather than the northern photoperiod. On the other hand, northern and northernmost populations did not differ between each other and grew faster in the northern rather than in the southern photoperiod. Field sampled adults did not differ in size across the studied regions.

**Conclusion/Significance:**

We found a significant differentiation in growth rate across latitudes and latitudinal difference in growth rate response to photoperiod. Importantly, growth responses measured at a single larval developmental stage in one season may not always generalize to other developmental stages or seasons.

## Introduction

Latitudinal changes in average temperature and in the length of the growth season is expected to have a great influence on size and age at metamorphosis [1], [2]. Lower average temperatures at higher latitudes will lead to a reduction in nearly all biological rates [3], including growth rates. Similarity, shorter growth seasons at higher latitudes will lead to a reduction in individuals’ growth potential. In an obligately univoltine species (i.e. that require one year to complete a generation), this is expected to result in a reduction in body size at maturity [4], giving rise to a decline in size with latitude, known as an inverse Bergmann’s cline. A reduction in size, however, is likely to have important fitness consequences for individual organisms as body size is an important predictor of individual’s fecundity, mating success and longevity [2] but see [5]–[8]. Several studies have demonstrated the existence of physiological mechanisms that can compensate for this negative latitudinal trend in growth potential. More specifically, populations that experience low temperatures or are under strong seasonal time constraints have often evolved higher routine growth rates, reducing or completely offsetting differences in final body size that would otherwise have been observed [9], [10]. However, variation in voltinism (the number of generations per year) will greatly alter these patterns.

Many temperate species of insects display plasticity in voltinism across latitudes, with longer generation lengths at high latitudes [2]. When maturation is delayed, selection for compensatory growth responses will be greatly reduced and high-latitude populations can actually become less time stressed than low-latitude populations despite living in a less favorable environment [11]. Because of this, latitudinal variation in voltinism has been suggested to explain why reduced growth rates and increased adult size with latitude is sometimes observed [12].

That there exists a fundamental fitness trade-off between an individual’s size and age at maturity is a central tenet of life history theory [4]. Consequently, whether individuals respond to selection imposed by latitudinal gradients through increasing growth rates or delaying maturation will depend on the relative costs and benefits of employing either strategy [13]. Because high growth rates are associated with fitness costs [14]–[16], different latitudinal populations are expected to evolve optimal, rather than maximal, growth rates and generation lengths [11]. Because of these trade-offs between age and size, there is an increasing awareness that we need to jointly consider temperature, seasonality, growth, development and the timing of reproduction in order to fully understand latitudinal variation in life history traits [17]–[19].

Despite the important role of temperature for nearly all aspects of ectotherm performance, temperature represents an unreliable seasonal cue, due to great within and between season variability [11]. Photoperiod, in contrast, provides a noise free, seasonal cue that many temperate organisms use to trigger and adjust rates of growth and development so as to maximize fitness under local conditions [20]. In many temperate zone insects, the onset of winter diapause is triggered by photoperiodic cues. Because the day length on a particular date during the growth season becomes longer with latitude [21] and because organisms generally cease to grow well in advance of the onset of adverse conditions, the critical day length for the initiations of winter diapause typically lengthens with latitude [21]. Just like growth and time to maturity, we would expect the timing of when individuals cease to grow will confer fitness consequences, and that populations at different latitudes will evolve locally optimal sensitivities to photoperiod [21], [22]. There are several reasons why time stress may cause selection on photoperiodic responses and why, conversely, photoperiodic responses can alter levels of time stress. First, when time stress is strong, reduced sensitivity to photoperiod may evolve (i.e. shorter day lengths are needed to induce diapause), allowing individuals to grow later into the season, at the cost of an increased risk of being exposed to deleteriously low temperatures. In contrast, because thermal variability is greater in high-latitude environments [23], natural selection may favor broader “safety margins” in high-latitude populations. If so, we would expect that high latitude populations will initiate diapause when days are relatively longer than low-latitude populations, to ensure that individuals enter diapause sufficiently early. Such a latitudinal trend in photoperiodic responses would cause an increase in the strength of seasonal time constraints in high-latitude environments. Importantly, in each of these cases, photoperiodic responses will modify the relationship between age and size across latitudes, motivating a joint consideration of these factors.

Surprisingly, there are relatively few studies that have examined how photoperiod influences growth in organisms that show flexible patterns of voltinism along latitudinal gradients. Because photoperiodic responses can completely override temperature responses, variation in this respect can potentially have large consequences. For example, manipulations of photoperiod at low temperatures have been shown to increase developmental rates as rapidly as an increase in temperature [24]. But the question of how photoperiod fits into the wider life history framework [25]–[28] remains unanswered. Photoperiodic responses are likely highly evolvable, as suggested by their important role in microevolutionary responses to climate change [29] as well as during invasion and range expansion [30]. This would suggest that photoperiodic responses are closely tied to other life history traits. Hence, populations originating from different geographic regions are likely to differ in photoperiodic responses. Investigating the interacting effects and possible trade-offs between photoperiodic responses, growth rate and age and size at maturity would thus help to further our understanding of how organisms adapt to seasonal environments. [12], [20], [31], how they are affected by and evolve in response to climate change [29] and during range expansions. The latter is especially important as many odonates are currently rapidly shifting their distributions towards higher latitudes [32]–[34].

The focal species that we study here, *Coenagrion johanssoni* (Wallengren, 1894), represents a northern damselfly species that shows latitudinal variation in voltinism and require more than one year to complete development throughout its range. There have been relatively few studies on life histories of odonates from high northern latitudes [26], [35], [36]. We examined in more detail if populations originating from different latitudes differ in routine growth rates, if a northern and southern type photoperiod have different effects on growth rates and if growth patterns differed before and after winter. The aims of this study were to (1) evaluate to what extent populations at different latitudes are differentiated with respect to growth rate and photoperiod responses, and to relate this variation to differences in seasonal time constraints and voltinism, and (2) to collect information on latitudinal variation in adult body size in the field, which will provide ancillary data on the potential strength of the size-age trade-off in nature.

Many different scenarios with respect to growth rate and photoperiodic responses are possible in this species but based on the current information, we predict the following: (1) there will be a significant difference in growth rate among populations. The northern and northernmost partivoltine (> 2 years/generation) populations will grow slower than the central semivoltine (2 years/generation) populations due to having one or several extra seasons available for development and growth. Many species of odonates and other insects respond to longer photoperiods by growing faster [21], [37]. In line with this, we predict that (2) all populations, but possibly to a different extent, will have higher growth rates under a northern photoperiod compared to a southern. Finally, we predict that (3) different photoperiods will have the same effect on growth rates both before and after the winter treatment. Adult body size of field collected damselflies is more difficult to predict as we do not have detailed information regarding changes in voltinism along the latitudinal cline [14]. However, our simple prediction is that (4) increases in growth rate in more time constrained populations will perfectly compensate for the decrease in generation time so that adult body size will show no variation across the studied regions [38].

## Methods

### Study Species

**Figure 1:**
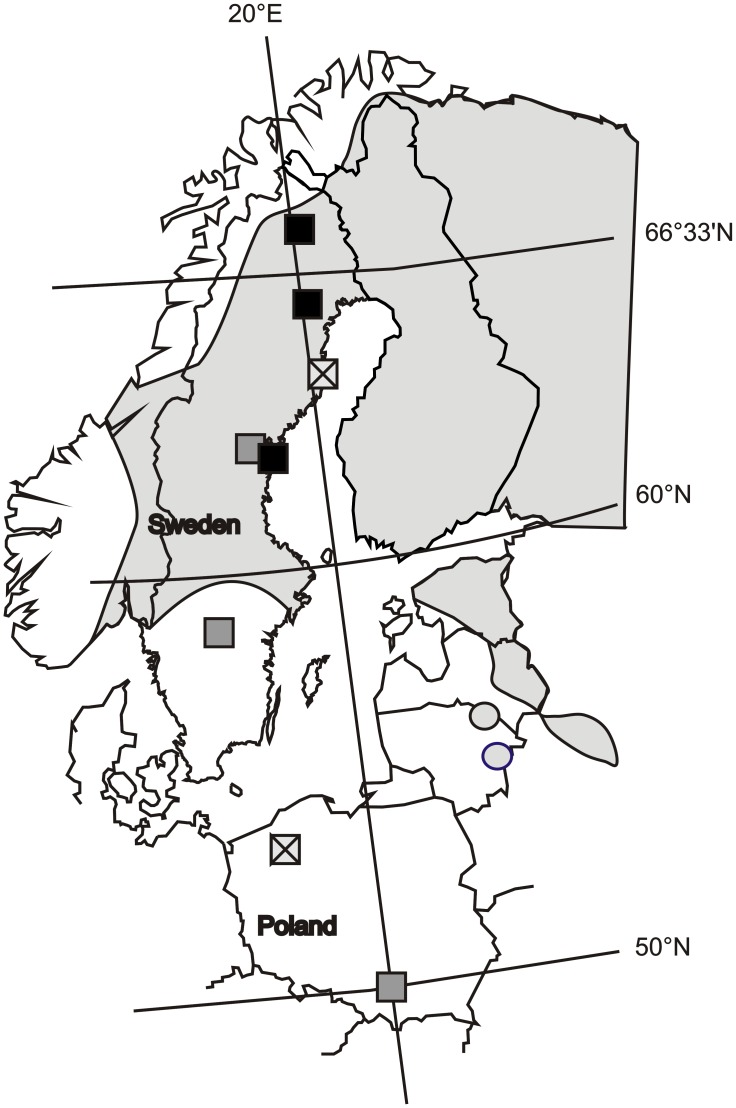
Map of northern Europe depicting the source populations and distribution of *C. johanssoni* and sampling sites in previous studies of other damselfly taxa (discussed in the text). The black boxes represent areas from which we sampled *C. johanssoni* (this study) grey boxes represent sampling sites of *C. puella* and *C. pulchellum* [46] and crossed boxes represent sampling sites of *L. sponsa* [22]. Gray shading shows the main area of European distribution of *C. johanssoni* based on [39].

*Coenagion johanssoni* is a northern species that is widespread but local in Northern Scandinavia, Russia, and very local in the Baltic States (Fig. 1) [39]. It is neither an endangered nor protected species. Larvae often develop in marshy bays or edges of lakes and bogs [39]. *C. johanssoni* overwinters in the larval stage [35], [39], [40]. No studies have explicitly investigated the life cycle length (voltinism) of this species [41]. However, studies on other ecologically similar species [35], [41] and judging by our own observations, *C. johanssoni* represents a Type 2 species [42] - a species that changes voltinism across latitudes. Based on field observations and computer simulations [43] we consider it most likely that it complete one generation per two years (semivoltine) in central parts of Sweden and one generation per three or even four years (partivoltine) in northern and northernmost parts of its Scandinavian distribution. The flight period stretches from the second half of June to the end of August ( [39]; authors’ unpublished data). There is no data available on the fertilization precedence in C. *johanssoni*. Last male sperm precedence (LMSP, i.e. the proportion of a female’s offspring that is sired by the last male with which she copulated) is generally believed to be high in damselflies, with studies of multiple damselfly species, including other *Coenagrion* species, reporting that levels of LMSP rarely falls below 95% ( [37] p 521). However, some studies have found LMSP to vary from 44% to >90% [44], [45] and it therefore appears likely that at least some egg clutches will represent a mix of full- and half-sibs.

### Field Collection

No specific permits were required for the described field studies. Egg collection and handling are described in detail in Śniegula and Johansson and Śniegula et al. [22], [46] and briefly summarized here. Egg clutches were produced by field-collecting copulating females, placing them in glass jars and presenting them with wet filter paper into which eggs were deposited within a few days. The females originated from three latitudinal populations, originating from central (collected between 10–13 July 2010), northern (20–22 July 2010), and northernmost Sweden (25–27 July 2010); Fig.1. In total, the investigated latitudinal range covers a 618 km long south - north gradient. Within each latitudinal population, several sub-populations were sampled. In central Sweden, three sub-populations were sampled (with six, four, and four females [families] collected at each site), in northern Sweden four sub-populations (five, three, three and three families from each site), and in the northernmost part of Sweden one sub-population (five families). A second sub-population in the northernmost part of Sweden was only sampled for measuring adult size. More than one sub-population was sampled within the central and northern regions in order to reduce the risk that maternal effects would have a directional effect on the outcome of the experiments (as females will have experienced a greater range of variation prior to the collection of eggs). Apart from measuring hatchling size, which may be related to a female’s condition, we did not evaluate possible maternal effects that could influence growth under laboratory conditions, but maternal effects have been found to be weak or non-existant with regards to larval development in related species [47], [48]. Possible maternal effects are further treated in the Discussion. The mean distance between sampling sites in each region was 6.3 km (range 0.7–15.5 km, SD = 6.1 km). All sampling sites were medium or small acidic lakes (mean size 8529 m^2^ SD = 9151.2 m^2^) with peat and *Sphagnum* vegetation along the shores and surrounded by boreal, coniferous forests. One lake in the central and northern region supported a fish population. We have no information about the fish status of the other lakes.

Once the females had laid eggs, the eggs were placed in Ziploc bags, placed in padded envelopes and sent by regular post to the laboratory in Kraków, Poland, within one or two days following ovipositioning. Eggs typically arrived within four or five days.

To get an estimate of how adult size varies in the field (providing information about what extent compensatory mechanisms succeed or fail to compensate for seasonal time constraints at different points along the latitudinal gradient), we collected a representative sample of adult damselflies at each visited site. The sample sizes differed somewhat between regions. In central Sweden, 20 males and females where collected from each of the three sites. In northern Sweden, 15, 17, 20 and 20 males and 6, 9, 17 and 19 females were collected from each of the four sites. In northernmost Sweden, 17 and 13 males and 7 and 7 females were collected from the two visited sites.

### Laboratory Experiment

The common garden experiment was run at the Institute of Nature Conservation, Polish Academy of Science, Kraków, Poland. The procedure was similar to that used in a previous experiment involving two congeneric species [46]. Eggs, and later larvae, were reared at a constant temperature of 22.5 ± 0.5°C. This temperature was chosen because (1) this is a temperature that larvae of the studied species will encounter in nature throughout the sampled latitudinal range [49], [50]; and (2) because larvae of *C. johanssoni* grow very slow at temperatures below 21.5°C [43].

**Figure 2:**
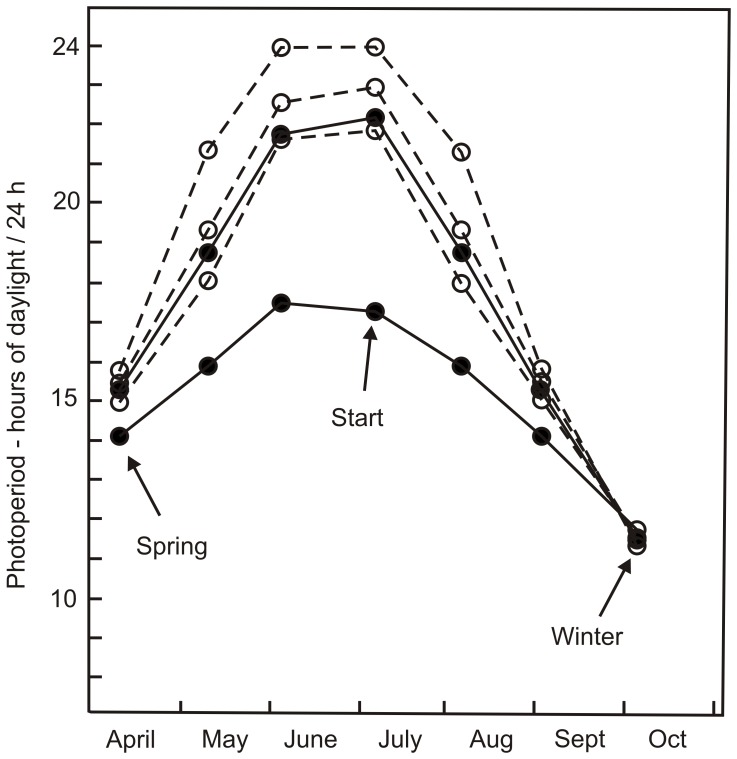
Progression of photoperiod (including half the Civil Twilights at sunrise and sunset) at five different latitudes between 52°N and 67°N. Filled circles connected by solid lines represent the two photoperiod treatments used during the experiment (northern, 64°N, above and southern, 52°N, below). Open circles connected by dashed lines represent photoperiods at latitudes where study damselflies were sampled (67°N, 66°N, and 62°N from the highest to the lowest lines, respectively). Arrows indicate dates of the start of the experiment (“Start”), the initiation of winter conditions (“Winter”), and the initiation of spring conditions (“Spring”).

Upon reaching the laboratory, all egg clutches was divided in half and placed in separate containers (12×8 cm, height 5 cm filled with approximately 200 ml of dechlorinated tap water) in one of two climate chambers. One chamber was set to mimic the natural progression of photoperiods at the latitude 52°N (henceforth called the “southern” photoperiod). The second chamber was set to mimic changes in photoperiod at the latitude 64°N (henceforth called the “northern” photoperiod) (Fig. 2). When the first eggs arrived, each of the two climate chambers was set to represent conditions on 10 July 2010 at each latitude (including half a period of morning and half a period of evening Civil Twilight [see 51]. This represented a light-dark (L:D) ratio of 17.15∶06.44 h in the southern photoperiod treatment group and a L:D of 22.06∶01.54 h in the northern photoperiod treatment group (Fig. 2). Thus, half of the embryos and half of the larvae from each region received the northern and southern photoperiod treatment, respectively. To follow the natural progression of day - night lengths, we changed the photoperiod regime in both chambers every Saturday. It is worth noting that organisms can be sensitive to changes in photoperiod in two ways: either by sensing rates of change in the relative length of days and night, or by being sensitive to the absolute length of days and nights [22], [42], [52]. Because differences in photoperiod across latitudes involves changes in both these factors (Fig. 2), differences in growth among treatment groups could result from either of these mechanisms in our experiment.

It is also important to note that the southern photoperiod treatment corresponds to conditions at a much lower latitude (52°N) than where the sampled populations originated from (the southernmost being central Swedish populations at 62°N). We chose to exaggerate differences in photoperiods between treatment groups for two reasons. Firstly, we hoped to magnify potential differences in photoperiodic responses. Secondly, we were interested in being able to compare with the results from earlier studies [22], [46]. However, there are several drawbacks with this design. In particular, it makes it hard to predict how individuals from different populations will interpret the meaning of the two photoperiodic treatments. Specifically, some populations will have experienced relatively similar photoperiods in nature to one of the treatment photoperiods, while for some populations both photoperiods will represent completely novel conditions that they may not “understand” (Fig. 2). We will discuss these difficulties further in the Discussion. We hope that by interpreting the responses of the studied populations to these photoperiods in relation to the responses of other species when exposed to identical conditions [46], differences and commonalities will emerge that can help understanding how species adapt to seasonal environments.

Eggs started hatching on 25 July and the majority of eggs had hatched on 15 August 2010 (about 2–4 weeks after eggs were collected). These represent the real dates. Hatching was generally synchronous. Five individual larvae (replicates) were chosen at random from each container (family) and used for the experiment. Each of these individuals was transferred to individual plastic containers (diameter 7 cm, height 4 cm, filled with dechlorinated water filled up to ca. 3.5 cm). At the start of the experiment, each photoperiod treatment consisted of a total of 165 larvae (14 central families x 5 replicates + 14 northern families x 5 replicates + 5 northernmost families x 5 replicates). Larvae were fed daily with 290 (SE: 15.4, n = 10) brine shrimp nauplii, *Artemia salina*, which is considered an *ad libitum* supply for damselfly larvae [53].

To estimate larval growth rates, each individual larva was photographed at regularly spaced time intervals. An identical approach was used in the previous experiment on damselfly larvae [46]. Pictures were taken at four times with an interval of 42 days, so that each individual was photographed at day 0, 42, 84 and 126 (with day 0 representing an individual’s hatching day). To estimate the size at hatching, we measured ten random hatchlings from each egg clutch in the high latitude photoperiod treatment. The mean head width of these individuals was then used as an estimate for the size of all individuals of the family. From day 42 and onwards, we measured the size of all individuals. Head width was used as an approximation of overall size in both laboratory-reared and field-collected individuals, as head width correlates closely with other measurements of body size and display less allometric variation than other traits [37]. Head width of laboratory grown larvae was estimated with the image analysis program ImageJ v. 1.36b. Adults collected in the field were measured with a digital calliper.

On 8 October 2010, when larvae were 54–75 days old, we initiated a two week long winter treatment. After two weeks of winter, on 23 October, we initiated the arrival of spring. The methods used to simulate winter and spring conditions were identical to those used previously [46]. To avoid a sudden change from a high to a low temperature, we first reduced the temperature to 16 ± 0.5°C on 8 October 2010, kept the photoperiod on and fed the larvae for a final time prior to winter. On 9 October 2010, the photoperiod was switched off, leaving larvae in total darkness, and the temperature was set at 5°C. Immediately prior to this day, L:D conditions were almost identical in the two treatments (L:D 11.45∶12.15 for the southern treatment and L:D 11.26∶12.34 for the northern treatment; Fig. 2). Larvae were not fed during the winter treatment. On 23 October, we initiated the arrival of spring by first switching on the lights. The photoperiod at that day was set to represent L:D conditions on 9 April 2011 at each of the two latitudes (representing L:D 14.04∶09.56 for the southern treatment and L:D 15.14∶08.46 for the northern treatment; Fig. 2). The temperature was changed to 16 ± 0.5°C, and feeding was resumed. Following this day, the photoperiod treatments returned to follow the natural progression of day lengths. After fifteen days at 16 ± 0.5°C, when photoperiods corresponded to conditions on 24 April, we raised the temperature to 22.5 ± 0.5°. Larvae where photographed twice before the winter and spring treatment (Day 0 and 42) and twice following the winter and spring treatment (Day 84 and 126). The size increase between photo-session 1 and 2 thus reflects summer/fall growth and the size increase between photo-session 3 and 4 reflects spring growth. The experiment ended when individuals reached the age of 126 days (excluding the 14 days of winter conditions).

### Calculating Summer/fall and Spring Growth Rates

To assess patterns of growth before and after the winter treatment, we calculated two different measures of growth rate, representing growth during summer/fall and spring, respectively. Because individuals grow sequentially through repeated molts, we chose to first model individual’s growth trajectories and then use the modeled trajectories to calculate individual growth rates at two points in time: one during summer/fall and one during spring. Because no growth occurs during winter, these two weeks were excluded so that an individual was considered to be of the same age at the start and end of the winter treatment. Individual growth trajectories were found to be very diverse. There was no mechanistic growth model which we tested (logistic, asymptotic etc.) that could be fitted for all individuals and we therefore modeled each individual’s growth trajectory using a more flexible, third-degree polynomial fitted to the data from the four measurement events:
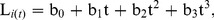
(1)where L_i(t)_ is the log head width of individual *i* at time t in mm*;* t is the time in days and b_0_-b_3_ are the estimated polynomial coefficients. Next, the growth rate of an individual in summer/fall and spring was calculated as the slope of Equation (1) at day 21 (i.e. half-way between the measured events 1 and 2). The growth rate in spring was calculated as the slope at day 105 (i.e. half-way between measurement events 3 and 4). The reason for adopting this approach rather than calculating growth rates between the fixed time intervals (e.g. day 42-day 0/42) was that we cannot regard the four measurement events as independent. Because insect growth occurs by first accumulating resources and then moulting, it is not justified to assume temporal independence of growth rates (as is done when calculating growth rates as the linear slope between fixed time intervals). Instead, by calculating growth rates from Equation (1) we take advantage of the valuable information contained in measurements from adjacent measurement events. Thus, our measurements of summer/fall growth reflect growth rates during the middle of summer/fall (day 21 in the experiment), based on all available information from hatching and up to (but not including) the winter treatment (i.e. day 0, 42 and 84). Similarly, our measurement of spring growth reflects growth rates in the middle of spring (day 105 in the experiment), incorporating all information from the onset of the winter treatment through spring (i.e. between days 42, 84 and 126). An aspect of growth that we cannot account for, though, is the likely size-mediated correlation between summer/fall and spring growth rates: individuals that attain a greater size in summer/fall will likely display decreased growth rates in spring due to this. This should be born in mind when interpreting these results.

### Statistical Analysis

A mixed-effect ANOVA was used to analyse latitudinal differences in size from field-collected adults. The region of origin, and sex were treated as fixed factors. Populations were treated as a random factor.

A one-way analysis of variance (ANOVA) was used to analyse latitudinal differences in hatchling size. Note that this analysis only includes hatchlings from eggs laid by females from different latitudinal populations that hatched in the northern photoperiod.

For the growth rate experiment, two separate mixed-effects ANOVAs were used to analyse the effects of photoperiod on the growth rate of individuals originating from different latitudinal populations during the summer/fall and the spring growth phase. The region of origin and photoperiodic treatment (northern, southern) were treated as fixed factors. Local sub-populations were treated as random factors, and families were nested within sub-populations. In the summer/fall analysis, the size at day 0 (i.e. at hatching) was initially included as a covariate as growth rates tends to decrease with size [54]. Similarly, size at day 84 was initially included as a covariate in the analysis of spring growth. Neither of these covariates was significant so they were excluded from the final models. We then also regressed hatchling size against growth rate separately for the summer/fall and spring growth to examine the relationship between hatchling size and the growth of later stages (a potential maternal effect). Note that because we only measured hatchling size in individuals hatched under a northern photoperiod, we only tested for association between hatchling size and later growth rate within this treatment group.

All analyses were performed in R 2.10.1 [55]. Mixed effects models were fitted using the function *lme* in the R package *nlme*. Significance levels of the main fixed effects were calculated on the basis of Wald *χ^2^* tests, using the function *Anova* in the R package *car.* This approach approximates traditional, hypothesis-tests in ANOVAs [56] but it should be noted that there is no general agreement on how to calculate degrees of freedoms in mixed-effects models [56]. Although population and, for the growth rate analyses, family were included as random effects in the mixed-effect models, we do not report significance test statistics for these as the experiment was not designed to detect differences at these levels.

## Results

### Field Sampled Adults

**Figure 3:**
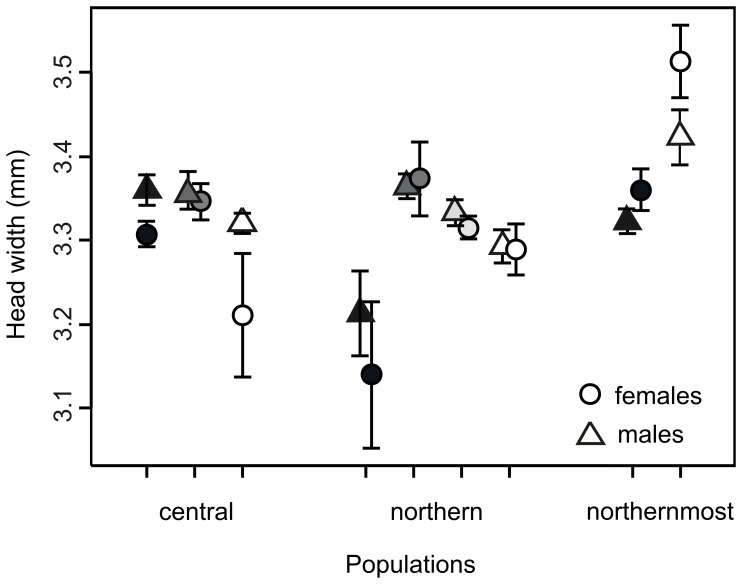
Head width of field collected adults of *C. johanssoni* originating from three different latitudinal regions across Sweden. Shading symbols indicate mean (95% CI) adult head width of different populations in each sampled region. Note that individuals from the second population sampled from the northernmost part of Sweden (marked with grey shading) were not included in the laboratory rearing experiment.

Adults collected at different latitudes showed no significant differences in size (Wald *χ^2^* = 0.52, d.f. = 2, *P = *0.77; Fig. 3). However, sexes differed significantly overall (Wald *χ^2 = ^*3.9, d.f. = 1, *P = *0.048), with males having on average 0.06±0.03 mm (±1SE) greater head width than females (Fig. 3). Although females tended to be somewhat bigger than males in the northernmost population, size differences between males and females did not change significantly across latitudes (Region x Sex interaction non-significant; Wald *χ^2 = ^*1.78, d.f. = 2, *P = *0.410).

### Size of Laboratory Hatchlings

**Figure 4:**
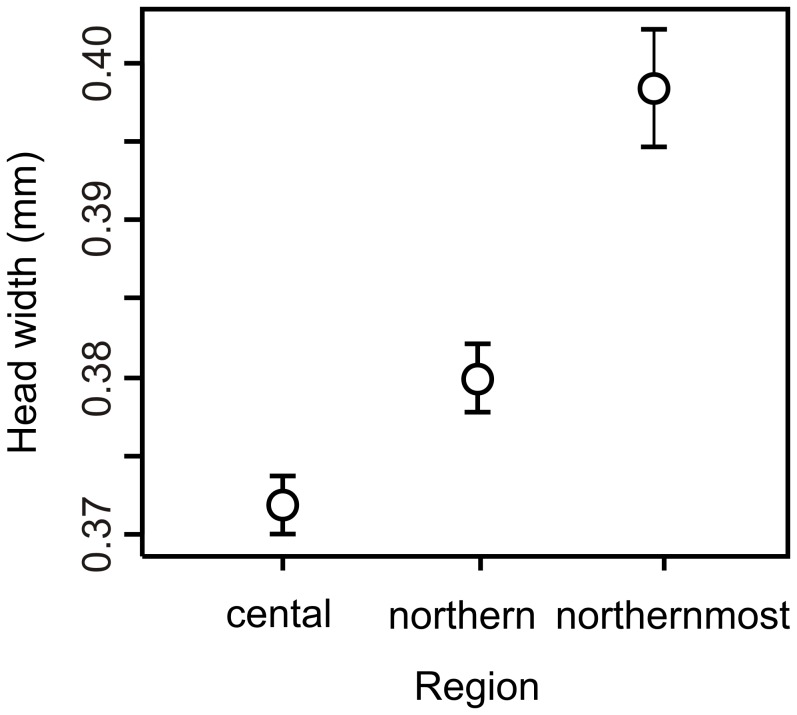
Head widths of newly hatched larvae of *C. johanssoni* when reared from eggs in the laboratory under a northern (64°N) photoperiod. Larvae originated from field-collected eggs laid by females sampled across three different regions in Sweden: the central, northern, and northernmost latitude regions. Symbols show mean (95% CI) values.

There was a significant difference between regions in the size of hatchlings that hatched in the northern photoperiod (*F*_2,290_ = 96.75, *P*<0.001). The biggest individuals hatched from the northernmost region clutches, intermediate size was noted from the northern region and the smallest size from the central region (Fig. 4).

### Summer/fall and Spring Growth Rates

**Figure 5:**
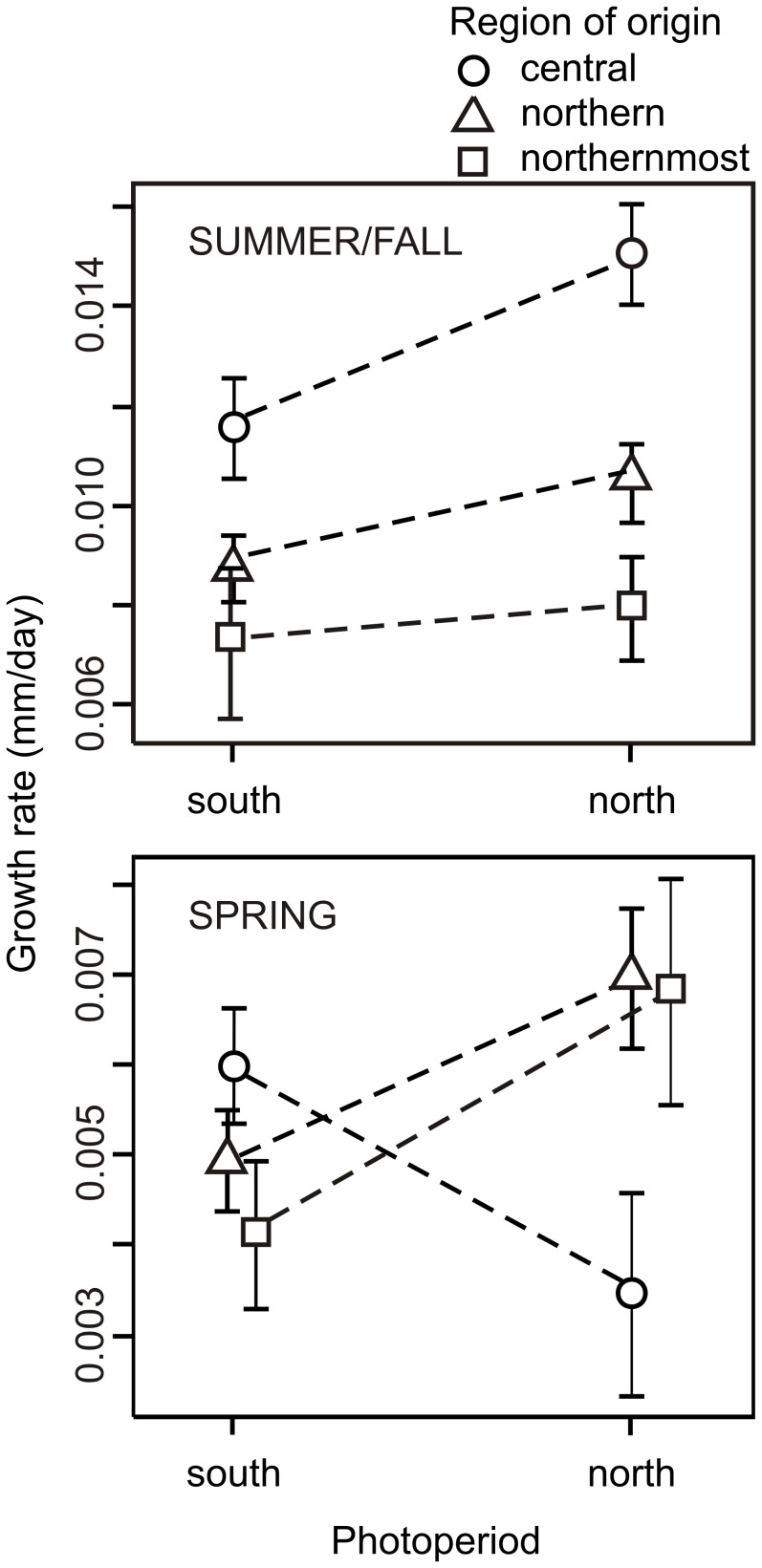
Mean larval growth rates across southern and northern latitude photoperiods. Larvae of *C. johanssoni* originated from three Swedish regions: central, northern, and northernmost latitudinal regions. Symbols show mean (95% CI) larval growth rates.

During the summer/fall growth phase, larvae displayed significantly higher growth rates under the northern photoperiod than under the southern photoperiod (Wald *χ^2^* = 47.33,d.f. = 1, *P*<0.001; Fig. 5). Larvae from the central region displayed significantly higher growth rates than larvae from the northern and northernmost regions, respectively (Wald *χ^2^* = 63.53, d.f. = 2, *P*<0.001; Fig. 5). The interaction between photoperiod and region was significant (Wald *χ^2^* = 11.23, d.f. = 2, *P = *0.003), indicating that regions differed in how photoperiods affected growth rates during the summer/fall phase (Fig. 5).

During the spring growth phase, there was no statistical difference in average growth rate between photoperiods (Wald *χ^2^* = 1.45, d.f. = 1, *P = *0.228) or regions (Wald *χ^2^* = 3.71, d.f. = 3, *P = *0.156). However, regions differed in how they responded to the two different photoperiods (Photoperiod x Region, Wald *χ^2^* = 39.68, d.f. = 2, *P*<0.001; Fig. 5). Individuals from the northern and northernmost populations displayed higher spring growth rates when reared under a northern compared to at southern photoperiod, whereas individuals from the central populations displayed higher spring growth rates when reared under the southern compared to the northern photoperiod (Fig. 5).

A linear regression showed a significant negative relationship between hatchling size and growth rate during summer/fall (*r* ^2 = ^0.45, *P*<0.001). The biggest hatchlings from the northernmost region grew slower than smaller hatchlings from the northern and central regions, respectively. In the spring growth, there was a significant positive relationship between hatchling size and growth rate (*r* ^2 = ^0.14, *P* = 0.001).

## Discussion

We detected significant differences in larval growth rates across latitudes of the northern damselfly *C. johanssoni.* During the summery/fall growth phase (i.e. prior to the winter treatment), growth rates decreased with latitude, being highest in larvae from the southernmost (i.e. central Swedish) populations and lowest in the northernmost population (Fig. 5). We also detected plasticity and variation in growth rate responses to changes in photoperiod. Changes in photoperiod had the same effect on all populations during summer/fall: individuals grew slower under a southern photoperiod compared to a northern photoperiod. During the spring growth phase, individuals from northern and northernmost Sweden showed the same responses as in summary/fall, but individuals from central Sweden showed the reversed response: they grew faster at a southern than at a northern photoperiod in spring (Fig. 5). With the exception of this latter observation, the observed patterns of plasticity (lower growth at the southern photoperiod) were opposite to what has previously been reported in two congeneric species [46].

Excepting central populations during spring (further discussed below), we consider the principal findings of this study (higher growth rates towards lower latitudes) to be an effect of a shorter life cycle compared to high-latitude populations [38] (Fig. 5). Because of the switch in voltinism that likely occurs between central and northern populations, the available growth period per generation actually becomes shorter in central populations. As a result, despite that they enjoy warmer temperatures and a longer growth season, central individuals will be under more severe time constraints and appears to have evolved a compensatory mechanism (higher growth rates) to ensure that individuals attain a greater size at emergence than they would have otherwise [2], [57], [58].

The size of field-collected adults did not differ across latitudes (Fig. 3). We predicted that increased growth rates in more seasonally time constrained regions (central Sweden) would perfectly compensate for the decrease in generation time. The growth patterns in summer/fall together with that we did not find a latitudinal trend in body size provide some support for the idea that higher routine growth may have evolved as a compensatory mechanism allowing individuals to emerge at the same size across latitudes, despite differences in seasonal time constraints. Whether latitudinal trends in body size are adaptive has been disputed for centuries [59] but most studies of latitudinal variation in body size have only considered field data, which confounds genetic and plastic differences in body size. By combining field data with laboratory data, we show that variation in growth rates may be involved in maintaining the same body size across latitudes. However, that central populations responded differently to photoperiod in spring (Fig. 5) strongly suggest that this interpretation is an oversimplification. Instead, individuals that grow fast during fall may not necessarily continue to grow fast the next year, suggesting that the relationship between growth rate and adult size is more complex. In addition, in the most exhaustive study to date of fitness under field conditions in a damselfly, Thompson et al. [60] found no correlation at all between adult size and fitness. Consequently, to emerge at a large size may not be the main reason for growing rapidly and Thompson et al. [60] suggested that being large may be more important during the early larval stages, as it can reduce the risk of intraguild predation. Because of these conflicting results, we are not able to draw any strong conclusions about whether body size variation is adaptive in this case. Studies of the timing of growth under natural conditions, preferably through transplantation experiments, would be very helpful for assessing how seasonal regulation of growth across latitudes contributes to clines (or lack thereof) in body size [18], [22].

Overall, however, we feel that these observations fit well with an increasing number of studies that have demonstrated that growth rate in relation to variation in generation length is a key element of adaptation to seasonal time constraints in a diverse assembly of animal taxa, including insects and damselflies [12], [46], [48], [61], [62]. These studies generally support that animals distributed along latitudinal gradients that impose seasonal constraints have evolved compensatory growth strategies, although it is not clear if adult size is an important factor. We did not explicitly quantify developmental rates, but the bulk of these data support the predictions from theoretical models that animals under time stress will speed up rates of both development and growth, effectively reducing variation in both size and age at metamorphosis across populations [63], [64].

Interpreting differences in photoperiodic responses are more challenging as the two photoperiod treatments represents a mix of relatively realistic (northern photoperiod) and completely novel (southern photoperiod) conditions to the studied populations. Nevertheless, that *C. johanssoni* responded in the complete opposite direction to these two photoperiods compared to two central European species of *Coenagrion* [46] gives some clues to how photoperiodic responses operates over larger geographic and taxonomic scales. We will first discuss growth patterns during summer/fall. In a previous study we found that *C. puella* and *C. pulchellum* from central Sweden and Poland (Fig. 1) had higher growth rates in a southern photoperiod compared to a northern photoperiod [46]. Our interpretation of this was that the short day lengths in the southern photoperiod were perceived as an indication that it was late in the season. Because both the previously studied species likely maintain a univoltine life cycle over their entire ranges, we argued that individuals would interpret short day lengths as a sign of strong time constraints, triggering rapid growth. High-latitude populations in addition had higher growth rates overall, supporting that a univoltine life cycle is the typical life history strategy in those species. That *C. johanssoni* did not display these patterns is likely to reflect the more complex latitudinal patterns in voltinism of this species. Because delayed reproduction (resulting in reduced time constraints) seems to be a typical life history response to seasonal time constraints in *C. johanssoni*, it may not respond to cues signalling time constraints by triggering an increased investment in growth. This strongly suggests that photoperiodic responses must be interpreted in relation to a species’ dominating life history strategy, and potentially to latitudinal variation in this respect. We will discuss the responses of *C. johanssoni* in this context next.

One explanation for the results in *C. johanssoni* is that the differences we observed in growth rate between the two treatments reflect latitudinal differences in the critical day length needed to initiate winter diapause. From this perspective, larvae will interpret the short day lengths at the southern photoperiod as a signal that it is late in the year, and consequently initiate winter diapause at an earlier stage, observed here as a reduction in growth rate at the southern photoperiod [26]. As we may expect high-latitude populations to initiate diapause at a longer critical day length than low-latitude populations [26], growth will cease earlier in high-latitude individuals compared to low-latitude individuals within each treatment group, as high-latitude individuals will encounter their critically short day length sooner than low-latitude individuals. The reduction in growth rates with latitude in both treatment groups matches this scenario (Fig. 5). If this interpretation is correct, the critical day for the induction of winter diapause must lie somewhere between 17 and 22 hours of day length in this species (Fig. 2). However, the same rank difference in growth rate between treatment groups was also evident already after 42 days, when the days were still relatively long and diapause initiation was unlikely to have happened. We are therefore not confident that the timing of diapause initiation provides a fully satisfactory explanation for the observed differences and we will explore several alternative explanations.

Adaptive phenotypic plasticity responses to photoperiod can evolve in response to selection acting over different hierarchical levels and spatial scales. It has been hypothesized that summer-active odonates have evolved a fixed photoperiodic response that allows individuals to continuously synchronize emergence along a latitudinal gradient [42]. If such an universal, adaptive photoperiod response would exist with regards to growth rates, individuals would be expected to respond similarity (and adaptively) to changes in photoperiod along a latitudinal gradient regardless of an individual’s latitudinal origin (and regardless if it had previously experienced that photoperiod). Evidence in support of such a latitudinal compensatory mechanism driven by photoperiod has been found in the damselfly *Lestes sponsa,* a strictly univoltine species that overwinter in the egg stage [22]. However, several lines of evidence argue against that such a mechanism is of any importance with regards to growth rates in *Coenagrion* species. The observation that growth rates increase under a northern photoperiod in *C. johanssoni* (Fig. 5) is in the direction predicted under such a scenario: growth rates will increase with latitude, which would counter the effects of low temperatures and growth seasons across latitudes. However, because of variation in voltinism and growth rates across latitudes (i.e. the observed overall decrease in growth rate with latitude is in the opposite direction to the photoperiodic response), it seems highly unlikely that this carries any adaptive significance in *C. johanssoni*. In the two central European *Coenagrion* species previously studied, such a latitudinal compensatory mechanism would seem to be more meaningful as it would aid in maintaining a univoltine life cycle over a large area. However, in those species the reversed photoperiodic response was observed [46].

Instead, explanations for variation in photoperiodic responses must perhaps be sought at a local scale and in relation to the prevailing life history strategy in each population. There is ample evidence that individuals have evolved a sufficient understanding of its native photoperiod to allow them to use photoperiodic cues for the timing of major life history events [20], [28]. In addition to this understanding of the native photoperiod, some phenotypic plasticity may evolve, allowing individuals to sense changes in photoperiod relative to their native photoperiod (i.e. an individual may sense if it is moved to the north or south of its native range and interpret this as an increase or decrease in seasonal time constraints, respectively). Such plastic responses may for example have been advantageous during range expansions, which nearly all temperate species have undergone following quaternary glaciations. We will make an attempt to interpret the present results from this perspective.

Under this scenario, central populations of *C. johanssoni* (62°N) would likely perceive the northern photoperiod (64°N) as a signal indicating increased time constraints compared to its native photoperiod. Furthermore, it seems reasonable to believe that the northern (65.5°N) and northernmost (66.5°N) populations would experience the northern photoperiod (64°N) as a signal of somewhat reduced seasonal constraints compared to their native photoperiod. However, to predict how either of these populations will perceive the southern photoperiod treatment becomes more speculative, as these represent completely novel conditions that it seems quite unlikely that they will have evolved an adaptive response to [11], [22], [31], [61], [62]. For the sake of this argument, we will assume that they have no *a priori* understanding of southern photoperiods.

Several alternative explanations for why larvae may grow slower at the southern photoperiod emerge from this interpretation, including: 1) an unknown photoperiod may not induce any compensatory growth response if rapid growth is dependent on receiving certain cues; 2) individuals may hesitate when exposed to unfamiliar conditions; 3) the shorter day lengths may cause individuals to be active during fewer hours each day, leading to a reduction in growth rates. These explanations are not mutually exclusive and none of them can be interpreted as an adaptive response to the southern photoperiod. If we assume that the low growth rates at the southern photoperiod emerge for the reasons stated above, and consequently reflect some base level of growth when no external stimuli induce rapid growth, it becomes more interesting to interpret relative changes in growth rates when individuals are exposed to the northern photoperiod. According to the above interpretation, central populations would perceive the northern photoperiod as an indication on an *increased* seasonal constraint compared to its native photoperiod. This may explain why central populations showed a greater difference in growth between northern and southern photoperiod (Fig. 5), which under this interpretation would represents compensatory growth induced by a northern photoperiod that represent *increased* seasonal constraint compared to its native photoperiod. Northern and northernmost populations, on the other hand, would perceive the northern photoperiod as indicative of *some* time constraints relative to the southern (uninterpretable) photoperiod, but it would rather indicate *less* time constraints relative to their native photoperiod. Under this interpretation, the smaller difference in growth between northern and southern photoperiods in northern and northernmost populations (Fig. 5) may represent a relatively weaker growth induction. An ideal experiment in which to test these ideas would be to grow larvae from each population at three different photoperiods, representing their native, a more southern and a more northern photoperiod. At present we tend to favour this latter explanation involving that individuals interpret photoperiods in relation to their native conditions as it can also explain responses of the two previously studied species [46], but clearly more research is needed to sort out how important absolute day lengths and rate of change in photoperiod are for regulating seasonal growth and development in species inhabiting temperate environments.

Responses to spring photoperiod differed significantly between regions and in comparison to summer/fall photoperiods (Fig. 5). Interestingly, individuals from central populations grew more rapidly under the southern than northern photoperiod in spring. A possible explanation for the slow spring growth of individuals from central populations under a northern photoperiod may lie in the large size these individuals attained prior to the winter treatment. Because absolute rates of growth tend to decrease with size, this may represent a consequence of this [54]. Alternatively, however, it may indicate that individuals respond differently to photoperiod cues depending on what size they have attained at a given time. In dragonflies, whether an individual will emerge in summer is determined during fall in the preceding season, based on the size individuals reach upon initiation of winter diapause [26], [40]. Because central populations had high growth rates during summer/fall under the northern photoperiod, they were bigger at the onset of winter than the other treatment groups and may therefore have exceeded this species’ critical size for next-year emergence. If so, this would have triggered a genetic program eventually leading up to them emerging during the following flying season. Even though at least one more moult will be required before emergence, these larvae will not be under strong time constraints. Consequently, a reduced investment in growth or a reduced sensitivity to photoperiod may be part of this genetic program.

For the other treatment groups, photoperiodic responses during spring showed somewhat similar patterns as in summer/fall. Also for these, two alternative explanations may apply. Either, individuals may interpret the short day lengths of the southern photoperiod as an indication that it is still winter so that they remain in diapause longer. Alternatively, the southern photoperiod has no direct meaning to these larvae and do not induce strong growth responses, whereas the northern photoperiod indicate mild time constraints and induce relatively stronger growth responses (see above). Note, however, that in spring (in contrast to in summer/fall), growth rates under the northern photoperiod did not differ between larvae from northern and northernmost populations (Fig. 5). Because larvae from the northernmost population were smaller than northern larvae when the winter started, they should be more strongly motivated to start to grow rapidly when spring arrives. We cannot draw any strong conclusions regarding these issues, but they raise interesting questions about how an individual’s size, and its prospects for future growth, may modify responses to photoperiodic cues.

That hatchling size differed between regions (increased with latitude) and was negatively correlated with growth rates during summer/fall but positively correlated with growth rates during spring is to our knowledge a novel finding in this study. We assessed variation in hatchling size and its effect on later growth as maternal effects may be carried over to offspring through hatchling size. Maternal effects are frequently assumed to have a negligible influence on the growth rate in damselfly larvae [18], [47], [48]. However, the direction of these changes was clearly not those predicted if hatchling size was carrying over maternal effects. We do not suggest that maternal effects are absent in this system and there are several other potential factors, for example egg size, egg volume or energy content that we did not consider. Also, maternal effects may be strong for other life history traits, and they have for example been shown to have a great influence on an offspring’s day of maturation in *C. puella* [65]. Rather, we strongly encourage further research on the potential importance of hatchling size in this system since we found a clear latitudinal pattern and an unexpected link to growth rate (Fig. 4). But since differences in hatchling size were so pronounced here, but evidently did not have any directional effect, we feel confident that maternal effects had only a limited influence on the growth patterns reported here.

In summary, growth during summer/fall suggested that different latitude populations of *C. johanssoni* display a variation of growth rates in response to increased time stress caused by shifts in voltinism. *C. johanssoni* responded to changes in photoperiod in the opposite direction to previously studied species, which may be due to that it displays variation in voltinism across its range. Finally, photoperiod responses differed between summer/fall and spring in central populations, suggesting that results from experiment carried out at a single ontogenetic stage and season may not be general to other stages, sizes or seasons.
